# Self‐Adaptive Graphdiyne/Sn Interface for High‐Performance Sodium Storage

**DOI:** 10.1002/advs.202401240

**Published:** 2024-05-10

**Authors:** Shujin Cheng, Zicheng Zuo, Yuliang Li

**Affiliations:** ^1^ Beijing National Laboratory for Molecular Sciences (BNLMS) CAS Research/Education Center for Excellence in Molecular Sciences Institute of Chemistry Chinese Academy of Sciences Beijing 100190 P. R. China; ^2^ Department of Chemistry University of Chinese Academy of Sciences Beijing 100049 P. R. China

**Keywords:** graphdiyne, interfacial protection, pulverization, sodium‐ion battery, tin anode

## Abstract

Efficiently reconciling the substantial volume strain with maintaining the stabilities of both interfacial protection and three‐dimensional (3D) conductive networks is a scientific and technical challenge in developing tin‐based anodes for sodium ion storage. To address this issue, a proof‐of‐concept self‐adaptive protection for the Sn anode is designed, taking advantage of the arbitrary substrate growth of graphdiyne. This protective layer, employing a flexible chain doping strategy, combines the benefits of 2D graphdiyne and linear chain structures to achieve 2D mechanical stability, electronic and ion conductions, ion selectivity, adequate elongation, and flexibility. It establishes close contact with the Sn particles and can adapt to dynamic size changes while effectively facilitating both electronic and ion transports. It successfully mitigates the detrimental effects of particle pulverization and coarsening induced by large‐volume changes. The as‐obtained Sn electrodes demonstrate exceptional stability, enduring 1800 cycles at a high current density of 2.5 A g^−1^. This strategy promises to address the general issues associated with large‐strain electrodes in next‐generation of high‐energy‐density batteries.

## Graphical abstract

A self‐adaptive interfacial protection is achieved for the Sn anode based‐on the 2D graphdiyne. It can adapt to the dynamic size changes while facilitating both electronic and ion transports. It effectively mitigates the detrimental effects of particle pulverization and coarsening induced by large‐volume changes. The lifespan of Sn for storing the Na^+^ is remarkably increased.

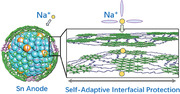

## 1 Introduction

Recent developments in sodium‐ion batteries (SIBs) have demonstrated their significant potential in replacing lithium‐ion batteries (LIBs) in many important application scenarios, such as grid‐level energy storage.^[^ ^1^, ^2^, ^3^ ^]^ This is predominantly attributed to the ample availability of resources, affordability, and appealing performance.^[^ ^4^, ^5^ ^]^ The low energy density is a significant obstacle for state‐of‐the‐art sodium‐ion batteries (SIBs), as it is limited by the intrinsic characteristics of sodium ions (Na^+^) that have a larger atomic weight and radius than Li^+^. Tremendous progress has been achieved in the development of cathode materials;^[^ ^6^, ^7^ ^]^ however, the advancement of anodes has been slow. The current hard carbon anode exhibits a limited capacity of 300 mAh g^−1^, which is insufficient for constructing high‐energy‐density SIBs.^[^ ^8^, ^9^ ^]^ Developing anodes that possess higher capacity and lower working plateaus is a critical strategy to complement the energy‐density gap between SIBs and LIBs.^[^ ^10^, ^11^, ^12^, ^13^, ^14^ ^]^

To date, extensive research efforts have been dedicated to the development of promising tin‐based anodes. This is because each Sn atom can alloy with 3.7 Na atoms resulting in a theoretical capacity of 846 mAh g^−1^ and the working voltage is lower than many alternatives.^[^ ^15^, ^16^, ^17^, ^18^ ^]^ However, the substantial volume expansion (420%) experienced during cycling leads to severe pulverization, coarsening, and repeated formation of the solid electrolyte interphase (SEI).^[^ ^19^, ^20^ ^]^ These factors result in rapid degradation and low Coulombic efficiency in the lifespan of sodium storage, thereby significantly undermining its advantages.^[^ ^21^ ^]^ Evidently, a significant contradiction arises between the substantial volume strain and the necessity to preserve interfacial protection and consecutive networks that facilitate both charge transportation and mechanical stability.^[^ ^22^ ^]^ Despite the implementation of various structural engineering strategies, such as porous frameworks, yolk‐shell structures, alloying, and surface coating, the issue persists as a formidable challenge.^[^ ^23^, ^24^, ^25^, ^26^, ^27^, ^28^ ^]^ Therefore, through targeted analysis, an optimal strategy should involve intelligent protection for Sn that can adapt to real‐time changes in size, while maintaining excellent electronic and ion conductivity and selective transport. This proof‐of‐concept protection has not yet been developed to address the dynamic issues of Sn anodes.

Graphdiyne (GDY) is a 2D porous carbon material that exhibits numerous intelligent properties.^[^ ^29^, ^30^, ^31^, ^32^, ^33^, ^34^ ^]^ Based on these properties, self‐expanding ion channels, electrochemical actuators, and mimicking efferent nerves have been developed in recent years.^[^ ^35^, ^36^, ^37^, ^38^ ^]^ The investigation of GDY's extended π‐conjugation, porous characteristics, and uneven electronic distribution has been conducted to protect the next‐generation electrode in batteries.^[^ ^39^, ^40^, ^41^, ^42^, ^43^, ^44^, ^45^ ^]^ These highlight the potential for addressing complex issues through various approaches, including the suppression of side reactions, the enhancement of interfacial selectivity, the facilitation of electronic and ion conduction, and the optimization of reaction kinetics.^[^ ^46^, ^47^ ^]^ These achievements demonstrate that GDY has prominent advantages in addressing the problems associated with Sn anodes. It possesses intelligent protection capabilities and can effectively fulfill the self‐adaptive function during the alloying/dealloying reaction.

Here, an intelligent interfacial protection based on the GDY is developed to improve the performance of Sn in storing the Na^+^. To demonstrate the proof‐of‐concept of intelligent protection, a flexible chain monomer is introduced to dope the GDY during the in situ conformal growth process on the Sn nanoparticles. The superiorities of the 2D GDY and 1D chain are effectively integrated into the Sn interface, resulting in the formation of a protective layer that possesses high modulus, mixed electron/ion conductivity, high elongation, rebound, and selective transportation. The interfacial protection layer can elongate and recover in real time in response to the swelling and shrinking of Sn nanoparticles. This allows for continuous and stable electron and ion transport, as well as maintaining intensive contact with the Sn nanoparticles throughout the charging and discharging process. Ascribing to intelligent protection and structural engineering, the detrimental effects of particle pulverization and coarsening on performance are effectively mitigated.

## 2 Results and Discussion

### 2.1 Material Characterization

**Scheme 1:**
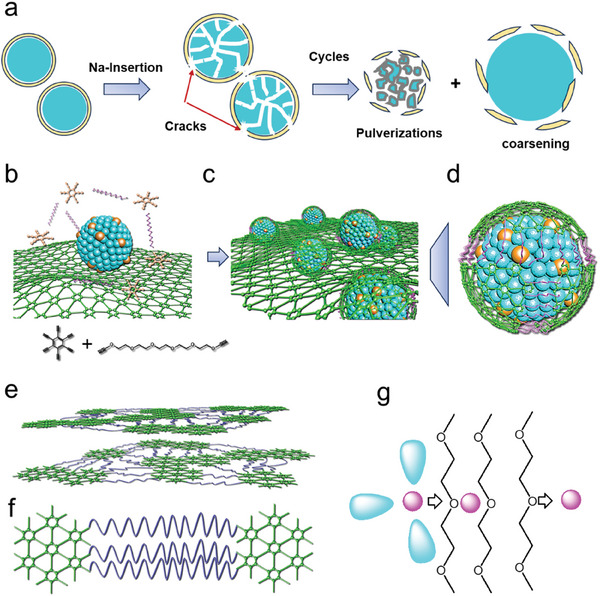
a) The degradation pathway of Sn anode in cycles; b–d) Schematic illustrations for showing the design and preparation of the GDY‐based self‐adaptive protection on the Sn particles; e,f) the molecular structure of protective layer; g) the interfacial desolvation of Na^+^ near the ether‐containing segment of the interface.

According to current coating techniques, such as carbon and inorganic coatings, the intensive protective layer on the Sn will experience rupture as a result of the significant volume change (420%). Such volume variation needs the reversible extension of protective layer up to 60%. **Scheme** **1**a briefly exhibits the decline in performance of the Sn anode. The presence of a hollow structure allows for volume change; however, loose contact diminishes reaction kinetics and interfacial protection. Thus, the processes of pulverization and coarsening are inevitable, resulting in a decrease in Coulombic efficiency due to the repeated formation of the SEI.^[^ ^48^ ^]^ Both pulverization and coarsening are reassembly phenomena in Sn electrode because the alloying/dealloying reaction cannot be spatially confined without physical barrier. To address this issue, a proof‐of‐concept of intelligent interfacial protection is presented in Scheme 1b, aiming to provide stability and strain recovery on the protective layer of the nanoparticles. The synthesis process involves introducing a flexible chain ending with acetylene groups to exceed the theoretical elongation of GDY (13.2%)^[^ ^49^ ^]^ to higher than 60%. In this coating layer (F‐GDY), GDY offers high 2D modulus, conductivity, and ion selectivity, while the 1D flexible chain provides a high strain, strain recovery, and an interfacial desolvation effect. Thus, the sodiation and desodiation reactions of Sn can be constantly confined by this protection layer. To ensure uniform growth of the GDY protection layer, partially alloyed Sn nanoparticles containing copper (Cu) elements are utilized as the active material (Figure S1, Supporting Information). The Cu element not only acts as a catalyst for the cross‐coupling reaction of GDY but also enhances inner conductivity and reaction kinetics^[^ ^28^ ^]^ during the volume swelling process, as depicted in Scheme 1c.

**Figure 1:**
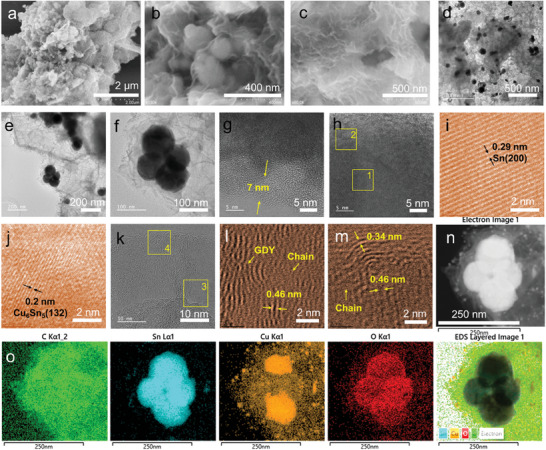
Morphological characterization of SnCu@F‐GDY. a–c) Typical scanning electron microscopy (SEM) images revealing the coating of F‐GDY on the SnCu nanoparticles; d) large‐scale TEM image of SnCu nanoparticles in the F‐GDY network; e,f) the magnification TEM image of the SnCu nanoparticles in F‐GDY; g,h) the high‐resolution TEM images of the SnCu@F‐GDY for showing the feature structure of the SnCu and F‐GDY; i,j) the ABSF‐filtered images of the selected areas in (h); k) the HRTEM image of the F‐GDY; l,m) the ABSF‐filtered images of the selected areas in (k); n,o) the corresponding elemental mapping images of SnCu@F‐GDY.

The morphologies of SnCu nanoparticles modified with GDY and F‐GDY are shown in **Figure** **1**, Figures S2, and S3 (Supporting Information). Before the modification, the SnCu nanoparticles had a spherical shape with an average diameter of ≈100 nm, as shown in Figure S1 (Supporting Information). After modification, the SnCu nanoparticles became encapsulated by F‐GDY and GDY nanosheets, forming the 3D continuous framework, as shown in Figure 1a–e, Figures S2–S4 (Supporting Information). As depicted in Figure 1g and Figure S5 (Supporting Information), it is evident that a consecutive and uniform layer of F‐GDY is conformally coated on the SnCu nanoparticles, which are ≈7 nm in thickness. The F‐GDY forms many wrinkles on the surface (Figure 1b,c), thereby enhancing the electronic transport pathways and providing 3D support to strengthen the mechanical stability of the SnCu@F‐GDY networks during the volume change process of SnCu nanoparticles. Different from the SnCu@GDY (Figure S2, Supporting Information), the wrinkles on the F‐GDY are less noticeable. The transmission electron microscopy (TEM) images provide additional evidence of the successful and uniform coating of SnCu nanoparticles with F‐GDY (Figure S5, Supporting Information). Furthermore, the TEM images also demonstrate that the SnCu nanoparticles can be effectively dispersed within the 3D F‐GDY networks (Figure 1d). Figure 1h shows the representative high‐resolution patterns of the SnCn nanoparticles containing both the Cu_6_Sn_5_ and Sn components. The ABSF‐filtered images of the selected areas in Figure 1h demonstrate the lattice spacing of 0.29 and 0.2 nm, which correspond to the Sn (200) (Figure 1i) and Cu_6_Sn_5_ (132) (Figure 1j) planes, respectively. For the F‐GDY, Figure 1k displays well‐organized patterns over a large area. The ABSF‐filtered images (Figure 1l,m) have two distinct spacing values, which can be attributed to the in‐plane lattice (0.46 nm) and the interlayer lattice (0.34 nm), respectively. The lattice pattern of Cu_6_Sn_5_ suggests that the Sn nanoparticles are partially alloyed with Cu. This is consistent with our structural engineering. The elemental mapping images (Figure 1n,o) provide clear evidence that the SnCu nanoparticles are effectively encapsulated by the GDY network, and the distribution of Cu elements on the nanoparticles is non‐uniform. After the growth of the F‐GDY, a portion of the Cu elements has been extracted from the Cu_6_Sn_5_, and subsequently dispersed onto the F‐GDY nanosheets. Importantly, the surface of the nanoparticles exhibits a notable enrichment of the oxygen element, suggesting a significant interaction between the ether chain and the SnCu nanoparticles during the growth of F‐GDY. This demonstrates the successful formation of an intelligent protection layer on the SnCu nanoparticles.

**Figure 2:**
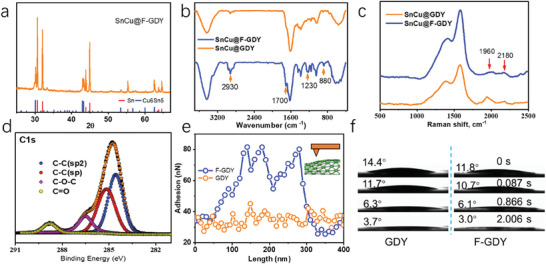
Structural information of the SnCu with and without the GDY modification. a) XRD patterns, b) FT‐IR, and c) Raman spectra of the as‐obtained SnCu anodes; d) high‐resolution XPS spectra of C1s; e) the adhesive power of the F‐GDY and GDY; f) spreading process of the electrolyte on the F‐GDY and GDY.

The as‐obtained materials are characterized to verify their chemical and physical properties. As shown in **Figures** **2**a and S6 (Supporting Information), the XRD patterns demonstrate that the SnCu@F‐GDY and SnCu@GDY contain both the Cu_6_Sn_5_ alloy and the Sn components, which is consistent with the bare SnCu. The Cu_6_Sn_5_ alloy component is utilized to initiate the growth of GDY and form the inner electron transport channels within the structure. Due to the relatively low GDY content (20%), the peaks originating from GDY are not discernible. Owing to the introduction of a flexible chain in the GDY, the FT‐IR shows obvious peaks from the ether chain. The peaks ≈2930 cm^−1^ are from the stretch of ─CH_2_─, and the peaks from 880 to 1230 cm^−1^ are from the ─C─O─ stretch. This indicates the successful introduction of the flexible chain into the GDY network. Raman spectra exhibit distinct peaks at 1940 and 2180 cm^−1^, which correspond to the characteristic Raman shift associated with the diyne linkage of the GDY network. To ascertain the composition of the GDY protection layer on the nanoparticles, XPS measurements were conducted. The high‐resolution XPS shows a remarkable difference in both the C1s and O1s (Figure 2d; Figures S7–S9, Supporting Information). Both the C1s and O1s spectra reveal the successful introduction of the flexible chain in the protection layer. The introduction of a flexible chain leads to significant changes in the interfacial properties, as demonstrated by the results of AFM and contact angle tests. According to the AFM test (Figure 2e; Figure S10, Supporting Information), it has been observed that the adhesive force of the F‐GDY is approximately twice as high as that of the GDY. The reason for this is that the AFM tip has a stronger interaction with the ether chain compared to the GDY. The contact angle of the electrolyte on the F‐GDY film is lower in comparison to that observed on the GDY film. As observed in Figure 2f, the spreading rate of the electrolyte drops on the F‐GDY film is found to be faster than that on the GDY film. This indicates that the introduction of an ether‐containing flexible chain leads to an enhancement in the solventphilic property of the interface. An enhanced solventphilicity can facilitate ion transportation across the interface, thereby accelerating the reaction kinetics.

### 2.2 Electrochemical Performance

**Figure 3:**
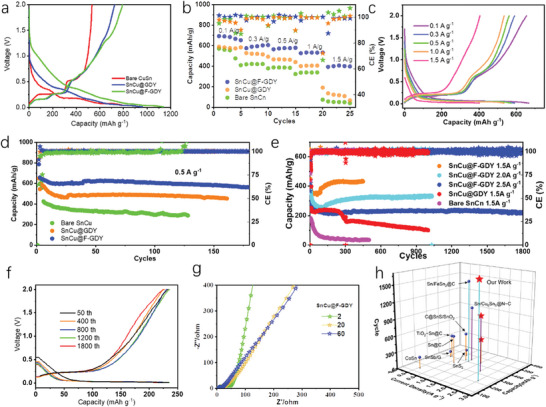
Electrochemical performance of samples. a) The first cycle charge/discharge curves of SnCu anodes before and after the interfacial modification; b) rate performance of the SnCu anodes; c) polarization curves of SnCu@F‐GDY at corresponding current density; d) the long‐term cyclability of SnCu anodes at current density of 0.5 A g^−1^; e) the comparison of long‐term stability of SnCu anodes at higher current density; f) the polarization curves of the SnCu@F‐GDY at diverse cycles; g) the electrochemical impedance spectra of SnCu@F‐GDY in cycling; h) performance comparison of the Sn‐based anodes for storing Na^+^.

To assess the influence of interfacial protection, the electrochemical performance of the SnCu anodes is measured. Seen from the first charge‐discharge curves of these samples, the charge/discharge polarization curves exhibit distinct multi‐step features with low working voltage plateaus (**Figure** **3**a). The introduction of interfacial protection has been shown to have a substantial impact on enhancing the specific capacity of materials. The SnCu@F‐GDY exhibits a remarkably high specific capacity of 800 mAh g^−1^, approaching the theoretical capacity of pure Sn. The enhanced capacity can be attributed to the decreased polarization during the alloy reaction with sodium, as well as the inherent storage capability of the GDY. After activation, the rate performance is measured at different current densities. As demonstrated in Figure 3b, the application of interfacial GDY modification can significantly enhance the rate performance of SnCu. Additionally, it is observed that SnCu@F‐GDY exhibits the highest capacity retention at all current densities. It can sustain a capacity of up to 580, and 400 mAh g^−1^ at 1 and 1.5 A g^−1^, respectively. In contrast, the SnCu@GDY and the bare SnCu can only have 400, and 320 mAh g^−1^ at 1 A g^−1^ and a capacity <180 mA g^−1^ at 1.5 A g^−1^, respectively. The optimized capacity retention at high current density can be attributed to the improved continuity of the network for both ion and electronic transport through the GDY modification. The highest performance of the SnCu@F‐GDY can be attributed to the synergistic effect of the flexible chain, which enhances the solvation/desolvation of Na^+^ across the interface. The typical polarization curves in Figure 3c and Figure S11 (Supporting Information) demonstrate that the SnCu anodes have low charge/discharge plateaus, with over 80% of the capacity being achieved at voltages below 1 V. This behavior is comparable to that of pure Sn, which is advantageous for the development of high‐energy‐density SIBs.

The investigation of interfacial protection is extended to examine its impact on the long‐term lifespan at different current densities. At a current density of 0.5 A g^−1^, the SnCu protected by GDY exhibits remarkable stability (Figure 3d). The SnCu@F‐GDY can still maintain a remarkable capacity retention of ≈560 mAh g^−1^ after 180 cycles. In comparison, the SnCu@GDY can retain ≈455 mAh g^−1^ after 160 cycles. As a control, the capacity of bare SnCn gradually decreases from 425 to 288 mAh g^−1^ after 125 cycles. At higher current densities, the performance showcases the benefits of the GDY modification (Figure 3e). It can be observed that the capacity of the bare SnCu at 1.5 A g^−1^ quickly declines from 190 to 20 mAh g^−1^ after 400 cycles. Protected by GDY, the SnCu@GDY has a remarkably improved stability as illustrated in Figure 3e. However, the capacity gradually decreases to 100 mAh g^−1^ after 1000 cycles at 1.5 A g^−1^. For the SnCu@F‐GDY, it demonstrates even better capacity retention during cycling (Figure 1e; Figure S12, Supporting Information). At 1.5 A g^−1^, it has a notable capacity of 428 mAh g^−1^ after undergoing 450 cycles. While the current density reaches high up to 2 A g^−1^, it demonstrates the capability to sustain a capacity of 330 mAh g^−1^ throughout 1000 cycles. When the current density is increased to 2.5 A g^−1^, the SnCu@F‐GDY anode exhibits impressive cycling stability. It maintains a capacity of 220 mAh g^−1^ with a stable charge/discharge plateau throughout a long‐term period of 1800 cycles. The ultra‐stable performance of the SnCu@F‐GDY can be attributed to the self‐adaptive protection of F‐GDY during the dynamic change process of the SnCu particles. This protection effectively ensures the continuity and stability of the channels for both electron and ion transport. The polarization curves obtained at various cycles, as shown in Figure 3f, provide additional evidence of low working voltage and exceptional stability of the electrodes at high rates throughout their lifespan. In Figure 3g and Figure S13 (Supporting Information), the EIS spectra demonstrate that the SnCu@F‐GDY exhibits the lowest and most stable electrochemical impedance during cycling. This suggests that robust interfacial protection is formed by the F‐GDY. Due to the implementation of this proof‐of‐concept interfacial protection, the SnCu@F‐GDY shows remarkable improvements in terms of capacity and long‐term stability compared to reported Sn‐based anodes.^[^ ^19^, ^25^, ^26^, ^27^, ^50^, ^51^, ^52^, ^53^ ^]^

**Figure 4:**
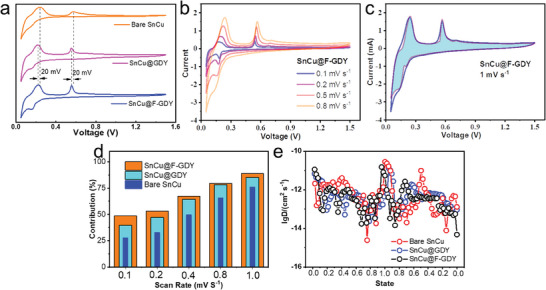
The kinetic properties of as‐prepared SnCu anodes. a) The CV curves of samples at the scan rate of 0.5 mV s^−1^; b) the CV curves of SnCu@F‐GDY at diverse scan rates; c) capacitive current contribution to the overall energy storage at 1 mV s^−1^; d) the capacitive contribution of SnCu anodes before and after the interfacial protection; e) The corresponding Na^+^ diffusion coefficients in the SnCu anodes.

The kinetic behaviors of the SnCu anodes are investigated to further confirm the influence of this intelligent GDY protection. The CV curves of these samples at a scan rate of 0.5 mV s^−1^ demonstrate that the presence of interfacial protection leads to a reduction of 20 mV in the reaction potential. Such a shift toward lower voltage is evident. Furthermore, the narrower peak widths and the noticeable reduction peaks at 0.18 V provide additional evidence of reduced polarization. Considering the high metallic conductivity of the SnCu anodes, we believe that the optimized kinetic behavior is mainly attributed to the enhanced 3D continuity of the conductive GDY network, as seen from the above SEM and TEM measurements. The CVs at diverse scan rates are presented in **Figures** **4**b and S14 (Supporting Information). It can be noticed that a higher scan rate in the bare SnCu results in a more pronounced peak shift, indicating increased polarization. According to the CVs, we conducted a further analysis to examine the kinetic contribution of the samples (Figure 4c; Figures S15 and S16, Supporting Information). The results in Figure 4d demonstrate that the GDY coating increases the capacitive contribution. The contribution is increased from 48.7% to 88.9% as the scan rate changes from 0.1 mV to 1 mV s^−1^. At 1 mV s^−1^, the SnCu@F‐GDY exhibits the highest value, while the bare SnCu and SnCu@GDY show values of 76% and 85%, respectively. The high capacitive contribution is the indication of high‐rate performance of the SnCu@F‐GDY. The GITT method is employed for the determination of diffusion coefficients (*D*) of Na^+^ in these systems. The *D*_Na+_ in the SnCu@F‐GDY (Figure 4e) and SnCu@GDY (Figure S17, Supporting Information) at different charge and discharge states are in the magnitude from 10^−13^ to 10^−12^ cm^2^ s^−1^, which is just comparable to that of the bare SnCu. In one specific cycle, these phenomena are reasonable and understandable because the SnCu anode has high metallic conductivity, and the system conductivity does not play a significant role in influencing ion diffusion. Besides, due to the absence of a stable interfacial protection layer on the bare SnCu, the desolvation reaction of Na^+^ on the interfacial protection of F‐GDY is another factor that limits the diffusion coefficient.

### 2.3 Protection Mechanism

**Figure 5:**
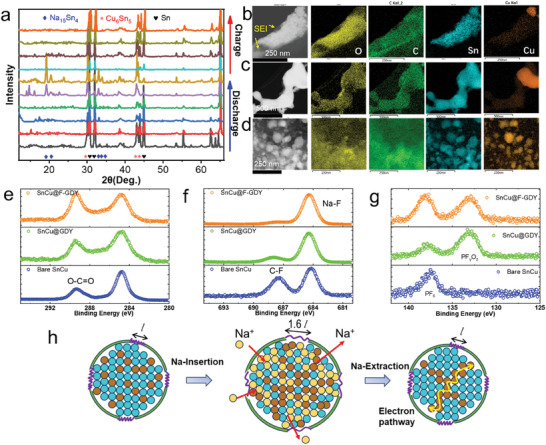
The degradation mechanism of the SnCu anode for storing the Na. a) the ex situ XRD measurement of the SnCu@F‐GDY in the first discharge and charge process; b) the pulverization and c) coarsening of the bare SnCu anode after 50 cycles; d) the structural variation of the SnCu@F‐GDY anode after 50 cycles; e–g) the high‐resolution XPS of the C1s, F1s, and P2p of the three SnCu anodes after 50 cycles, respectively; h) the illustration for showing possible mechanism of the high‐performance SnCu@F‐GDY anode.

The ex situ XRD of bare SnCu (Figure S18, Supporting Information) and SnCu@F‐GDY during the first charge/discharge cycle was conducted to investigate the reaction mechanism. As shown in **Figure** **5**a, during the sodiation reaction, the intensive peaks of Sn particles gradually diminish until they completely vanish, with the apparent final production of Na_15_Sn_4_. This observation indicates that the Sn is alloyed with Na, and each Sn atom is capable to capture 3.75 Na atoms. In the desodiation reaction, the Sn patterns reemerge, indicating the reversibility of the reaction. The presence of Cu_6_Sn_5_ peaks throughout the charge and discharge process suggests that Cu_6_Sn_5_ can act as a consistent pathway for electronic transport in the heterostructure. Comparing with the ex situ XRD of bare SnCu, the Cu_6_Sn_5_ is consistent in the charge/discharge process, and it can be found that the F‐GDY coating strategy shows no impacts on the alloying reaction mechanism of the electrode. Additionally, an analysis is conducted to characterize the SnCu electrode after cycling to examine the factors contributing to the performance improvement. As seen in Figure 5b,c and Figure S19 (Supporting Information), the pulverization and coarsening of particles coexist in the bare SnCu anode. Without interfacial confinement, it is evident that the SnCu nanoparticles undergo fragmentation, resulting in the formation of significantly smaller particles. Additionally, these particles become enveloped by a thick SEI. During this period, the Cu element is enriched clearly on some specific particles. Besides, the coarsening of SnCu nanoparticles is noteworthy, as shown in Figure 5c, and the segregation phenomenon of the Cu element is more significant. Such two phenomena occurring within the structure produce constant and significant harm to the connectivity of conductive networks and the stability of interfacial compositions, consequently resulting in a rapid deterioration in performance. Although coarsening and pulverization can be suppressed to some extent under the protection of GDY, coarsening can still occur once the GDY protection layer is broken due to repeated high‐volume swelling (Figure S20, Supporting Information). The reason for this is that a short theoretical elongation of the conformal GDY (13.2%) on the SnCu nanoparticles is insufficient to meet the requirements for synchronous changes in response to the substantial volume strain (60%). Impressively, these structural degradations can be effectively mitigated by employing the F‐GDY as a protective layer for the SnCu particles. This observation is demonstrated evidently by the corresponding images in Figure 5d and Figure S21 (Supporting Information). Due to the sufficient elongation of the F‐GDY and the synchronous extension and contraction of the conformal F‐GDY on the SnCu nanoparticles, the F‐GDY protection can physically prevent the coarsening of the SnCu nanoparticles, even when they are much closer to each other. Meanwhile, the uneven enrichment of the Cu element is inhibited clearly. The uniform distribution of Cu elements on the SnCu nanoparticles demonstrates that Cu can consistently facilitate the improvement of internal charge transfer within the particles. This is beneficial for promoting the reaction kinetics of SnCu under high charge/discharge current density. Both pulverization and coarsening can induce irreversible damage to the SEI, leading to the formation of a new SEI on the freshly exposed interface. This phenomenon causes significant variations in the morphologies of electrodes (Figure S22, Supporting Information). Under the protection, the SnCu@F‐GDY and SnCu@GDY have smoother and more continuous morphologies (Figures S23 and S24, Supporting Information) compared to the bare SnCu, suggesting the presence of a stable SEI supported by the GDY and F‐GDY. The interfacial compositions are further studied by the XPS, as shown in Figure 5e,f and Figure S25 (Supporting Information). Figure 5e–g demonstrates that the inorganic components of the CO_3_^−^, F^−^, and PF_y_O_z_ are formed as the SEI on the Sn anode, and the GDY and F‐GDY can enrich the contents of CO_3_ and F^−^. Among them, the SEI on SnCu@F‐GDY has the highest inorganic contents of Na_2_CO_3_ and NaF, advantageous for the inhabitation of interfacial side reactions. According to the abovementioned observations, the impact of this proof‐of‐concept interfacial protection on the SnCu anode can be schematically illustrated in Figure 5h.

## 3 Conclusion

In conclusion, a self‐adaptive interfacial protection based on the GDY is developed to address the strain‐induced issues of the Sn anode. This proof‐of‐concept protective layer applied to the Sn anode demonstrates the ability to elongate and recover in real‐time, effectively accommodating the swelling and shrinking of Sn nanoparticles. As a result, continuous and uninterrupted contact with the Sn nanoparticles is maintained throughout the dynamic process. It can preserve the exceptional 2D benefits of GDY in terms of mixed electron/ion conductivity, selective transportation, and high modulus. This strategy strengthens the continuousness and stability of the electron, and ion transport network, as well as the interfacial structure. Due to its robust protection, the structural and interfacial reconstruction resulting from the repeated pulverization and coarsening of the Sn anode are effectively suppressed. This allows for a high rate and long lifespan. This self‐adaptive protection shows general significance for solving interfacial issues in electrodes with large‐volume strain.

## Conflict of Interest

The authors declare no conflict of interest.

## Supporting information

Supporting Information

## Data Availability

The data that support the findings of this study are available from the corresponding author upon reasonable request.
