# Supplementary material for: Self‐Adaptive Graphdiyne/Sn Interface for High‐Performance Sodium Storage
Source: Adv Sci (Weinh). 2024 May 10;11(28):2401240. doi: 10.1002/advs.202401240 (PMC11267299; doi:10.1002/advs.202401240)
Supplement: Supplementary file 1 — Supporting Information [file ADVS-11-2401240-s001.pdf]

ADVANCED SCIENCE
Open Access

Supporting Information

for *Adv. Sci.*, DOI 10.1002/advs.202401240

Self-Adaptive Graphdiyne/Sn Interface for High-Performance Sodium Storage

Shujin Cheng, Zicheng Zuo and Yuliang Li**

Self-Adaptive Graphdiyne/Sn Interface for High-Performance Sodium Storage

Shujin Cheng [1,2], Zicheng Zuo [1*], Yuliang Li [1,2*]

1. Beijing National Laboratory for Molecular Sciences (BNLMS), CAS Research/Education Center for Excellence in Molecular Sciences, Institute of Chemistry, Chinese Academy of Sciences, Beijing 100190, P. R. China

2. Department of Chemistry, University of Chinese Academy of Sciences, Beijing, 100049, P. R. China

* Corresponding author. E-mail: zuozic@iccas.ac.cn; ylli@iccas.ac.cn

Material

The flexible chain of bis-propargyl-PEG4 and other chemical agents are all commercially available from Energy Chemical and the SnCu nanoparticles are from the Shanghai Chaowei Nanotechnological Co.. The SnCu nanoparticles content 15% atomic ratio of Cu elements, and the diameter is around 80 nm. All these agents were used directly without any further treatments. The monomer of HEB for growth of GDY is synthesized according to our previous method [1].

Preparation of the samples

For preparing the graphidyne-protected SnCu nanoparticles, 500 mg SnCu sample is first dispersed in 10 mL DMF by ultrasonication and the monomer of HEB (200 mg) is dissolved in 10 mL DMF, respectively. The use of the Cu element in the SnCu is for catalyzing the in-situ growth of graphdiyne on the SnCu nanoparticles. Then, the above SnCu solution and HEB solution are dropped into 150 mL diethyl ether solution under stirring. After 5 min, 10 mL pyridine is added into above reaction solution. This reaction solution is kept at room temperature under stirring for 3 days. After reaction, the sample of SnCu@GDY is collected by filtration and washed by the ethanol and acetone for three times, respectively. Subsequently, the as-obtained samples are thermally treated at 120 °C in a tube furnace for 2 hours in present of nitrogen gas. For preparing the

SnCu@F-GDY, the HEB (150 mg) and bis-propargyl-PEG4 (50 mg) are added instead of HEB only for the construction of F-GDY layer on the SnCu nanoparticles. Other procedures are similar with that for SnCu@GDY. For the growth of GDY and F-GDY film on the Cu foil, the Cu foil washed by the diluted HCl solution is immersed in 5 ml above corresponding solutions and the growth reaction is kept for 3 days in a sealed bottle.

Material Characterization

The XPS data are carried out on the Thermo Scientific ESCALab 250Xi using 200 W monochromated Al Kα radiation. A Renishaw-2000 Raman spectrometer with an excitation wavelength at 473 nm from an Ar laser is used to test the Raman spectra of samples. XRD patterns are recorded using an Empyrean diffractometer. SEM measurements are performed on a Hitachi Model S-4800. The TEM and HRTEM measurements are performed on JEM-2100F with an accelerating voltage of 200 kV.

Electrochemical measurement

For characterizing the electrochemical performance, the NMP slurry containing the samples, carbon black, and PTFE with the mass ratio of 8:1:1 is coated on the Cu foil by the doctor blade. After dying in the vacuum oven at 80 °C for 24 hours, the samples are cut into round pieces for the electrochemical testing. The mass loading is about 1.2 mg cm^{-2}. The electrolyte used in the half-cell tests is 1M $NaPF_6$ in DEGDME. The cell is assembled in the glove box. The XRD patterns of the electrode at different charge/discharge states are obtained using the ex-situ method. The cells are disassembled in the glove box, and the as-obtained electrodes are washed with pure DEGDME. After then, the electrodes are measured by the XRD instrument. Cyclic voltammograms at various sweeping rates and electrochemical impedance spectra (EIS) are recorded using an AutoLab apparatus. EIS are performed using a sinusoidal signal with an amplitude of 10 mV over frequencies in the range from 100 kHz to 0.1 Hz.

Statistical Analysis

XRD, HRTEM and XPS data were analyzed using Jade software, Digital Micrography software and Avantage software. Cyclic voltammetry and Galvanostatic

charge–discharge were analyzed by CH Instruments electrochemical software and LAND Dt software. The Galvanostatic intermittent titration technique and electrochemical impedance spectroscopy were analyzed by AutoLab and ZView softwares, respectively. Analyzed data was drawn using OriginPro software and merged with Adobe Photoshop software.

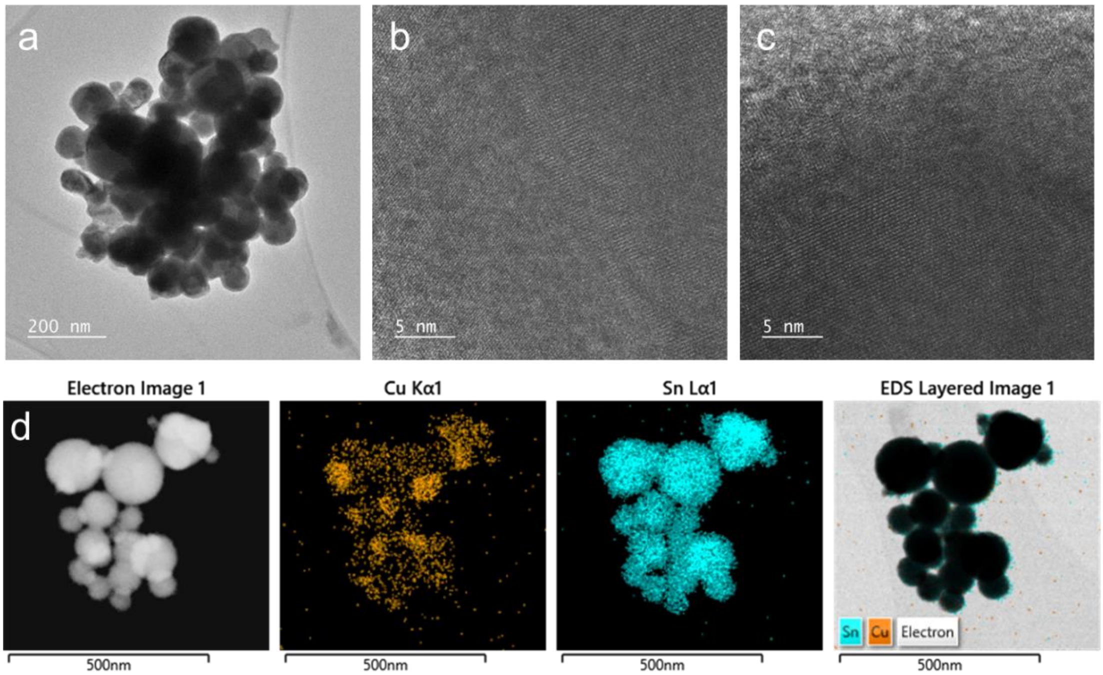

Figure S1. a) TEM images and b-c) the HR-TEM images of the bare SnCu nanoparticles; d) the elemental mapping images of the bare SnCu nanoparticles.

The HR-TEM images reveal the lattice patterns from the Sn and Cu_6Sn_5. The elemental distribution images also shown the SnCu nanoparticles are formed by the components of Sn and Cu_6Sn_5. The Cu element is used to catalyze the growth of GDY and tune the inner reaction kinetic of the nanoparticles in storing the Na.

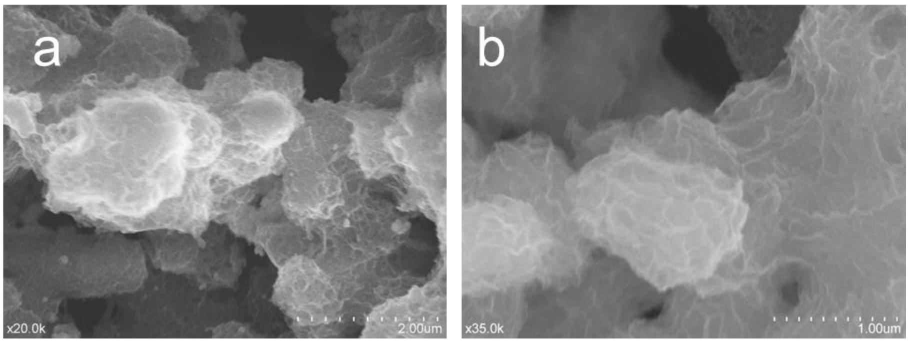

Figure S2. SEM images of the SnCu@GDY.

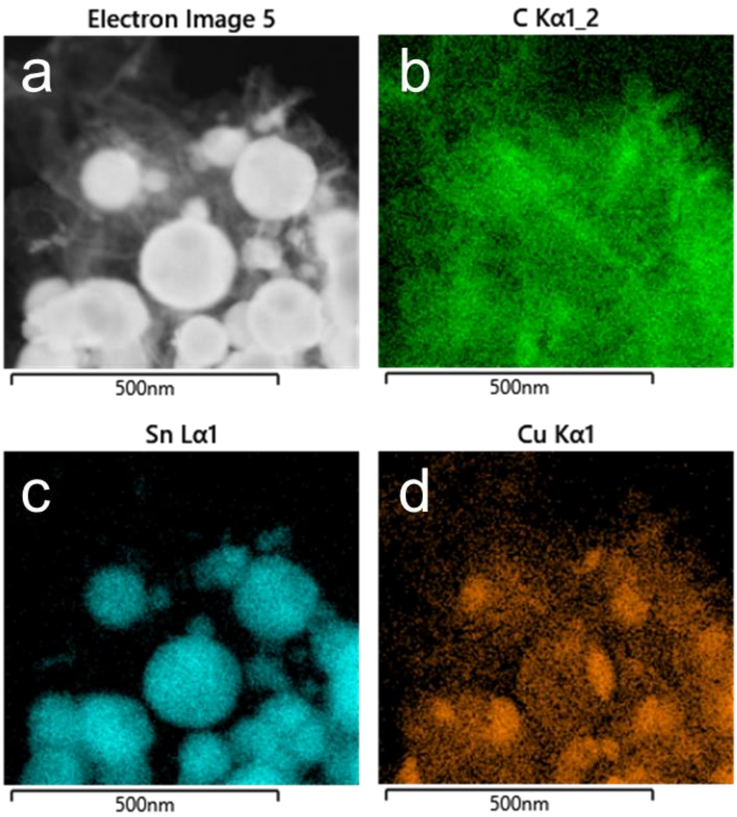

Figure S3. The elemental mapping of the SnCu@GDY.

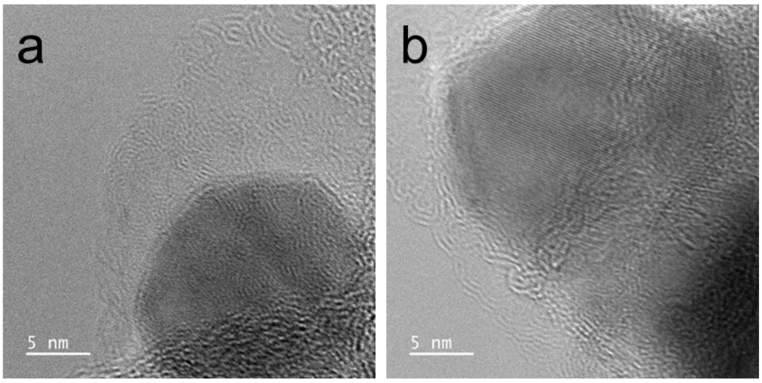

Figure S4. The high-resolution TEM images of the SuCu@GDY.

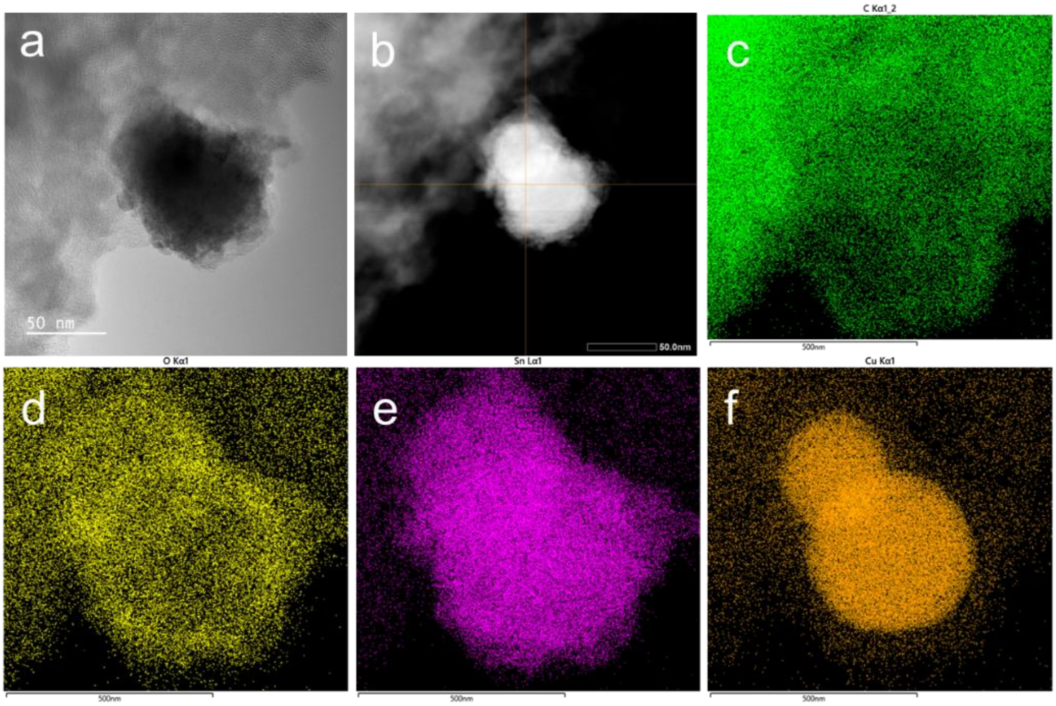

Figure S5. Elemental mapping images of the SnCu@F-GDY.

The elemental mapping images of SnCu@F-GDY shows the successful formation of the carbon coating layer containing the flexible chain on the SnCu nanoparticles.

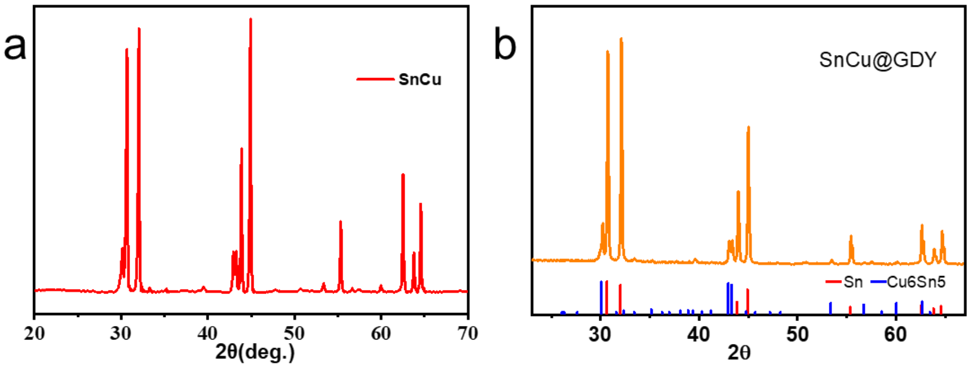

Figure S6. XRD patterns of the a) commercial SnCu and b) SnCu@GDY.

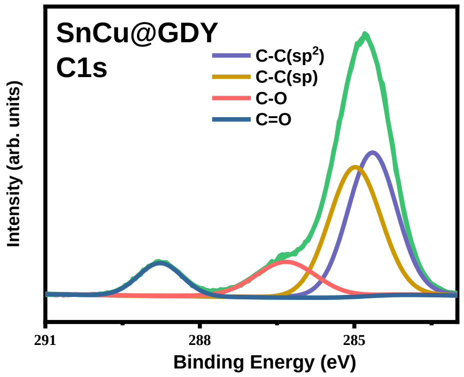

Figure S7. High-resolution XPS of the C1s of SnCu@GDY.

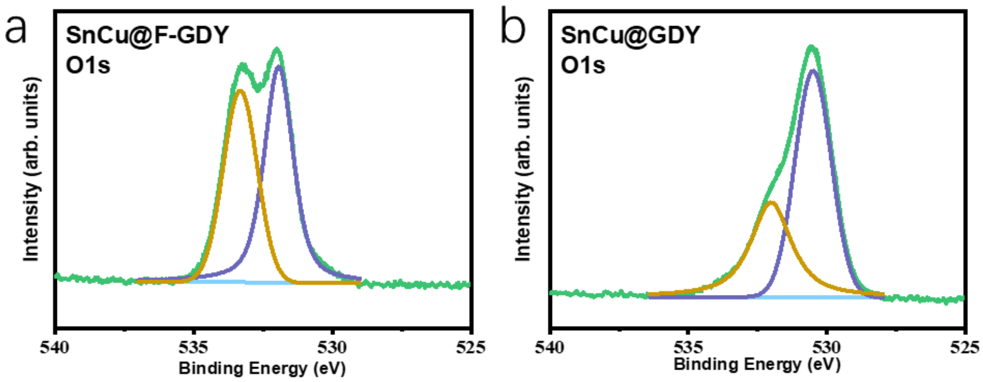

Figure S8. High-resolution XPS of the O1s.

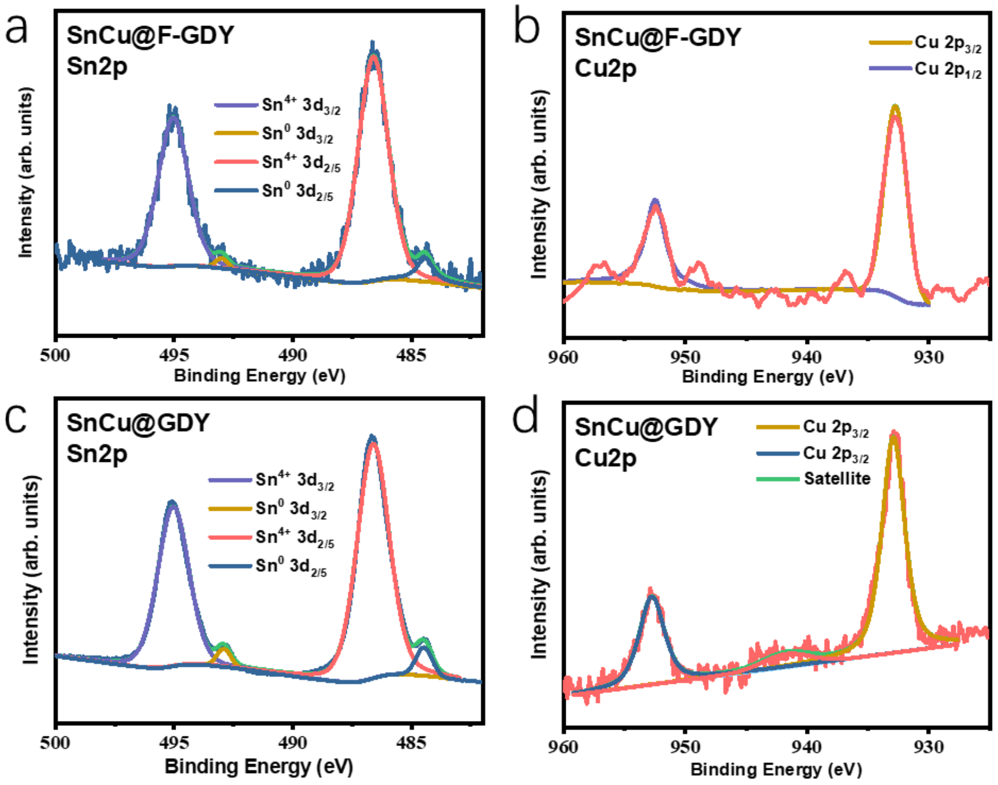

Figure S9. High-resolution XPS of the Sn2p and Cu2p of the SnCu@F-GDY and SnCu@GDY.

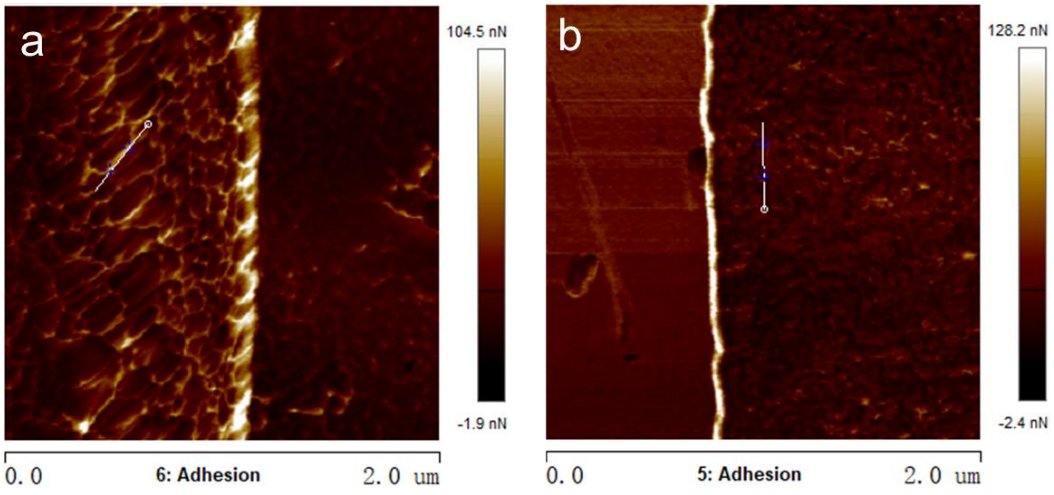

Figure S10. The AFM images of (a) F-GDY and (b) GDY film obtained under adhesive model. The typical adhesive curves obtained from the white lines in images are shown in Fig. 2e.

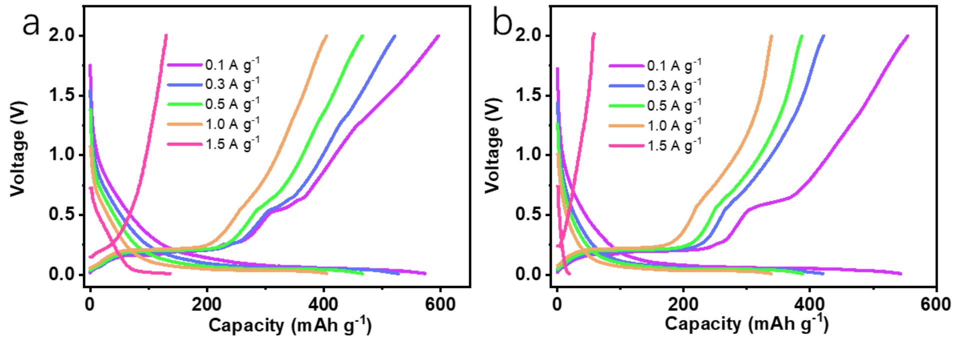

Figure S11. The polarization curves of SnCu@GDY and bare SnCu at diverse rates.

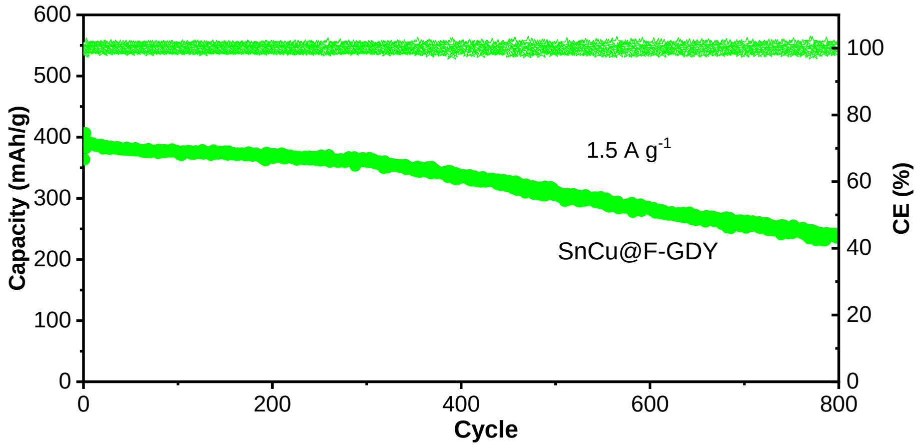

Figure S12. The long-term cycle of SnCu@F-GDY with a high loading of 2.4 mg cm^{-2}.

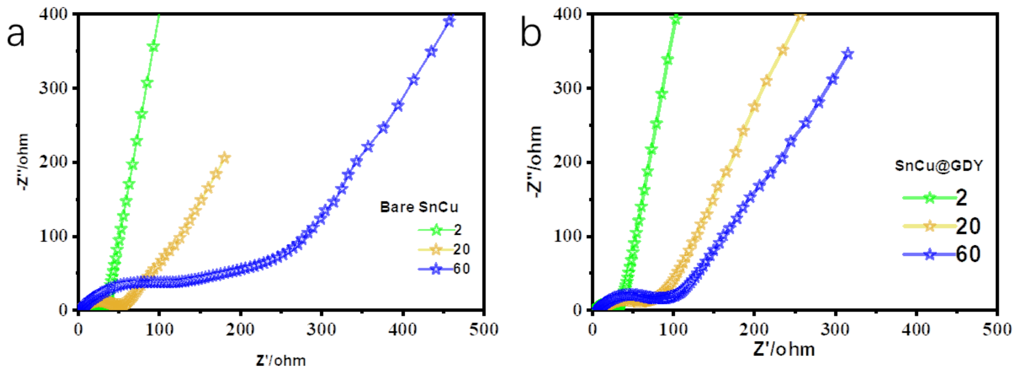

Figure S13. The electrochemical impedance spectra of the bare SnCu and SnCu@GDY at in the cycles.

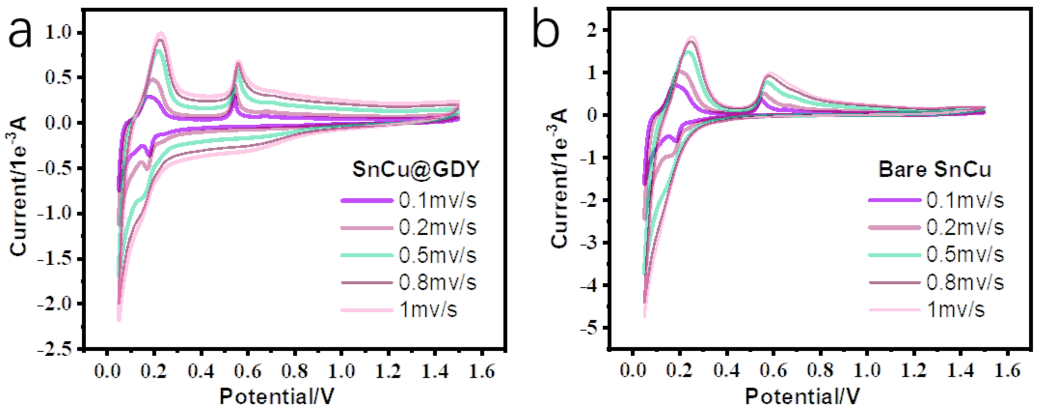

Figure S14. The CV curves of the a) SnCu@GDY and b) bare SnCu at diverse scanning rates.

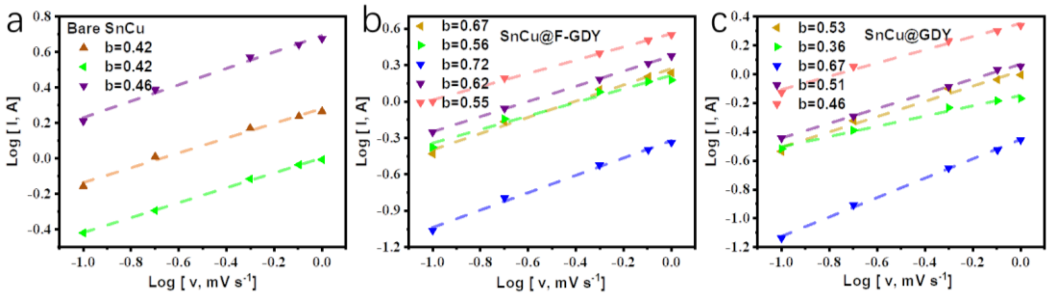

Figure S15. The relationship between the peak currents and sweep rates from the samples.

The high slope of these lines in SnCu@F-GDY and SnCu@GDY demonstrates that GDY protection increased the the surface-controlled Na^+ diffusion, beneficial for improving the kinetic performance.

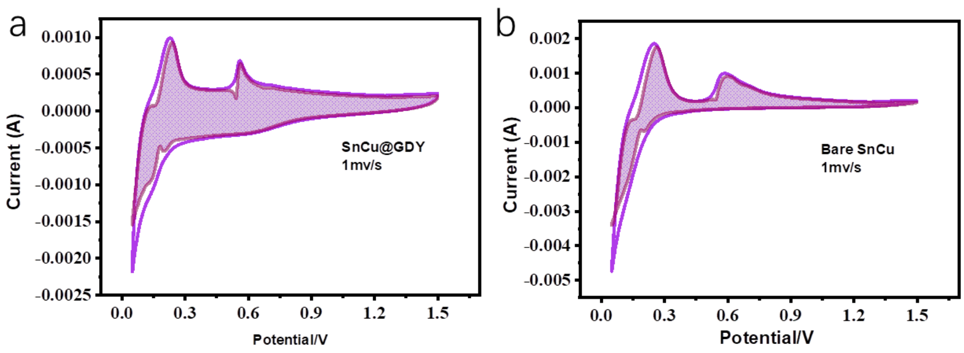

Figure S16. The capacitive current contribution to the overall energy storage of a)

SnCu@GDY and b) bare SnCu at 1 mV s^{-1}.

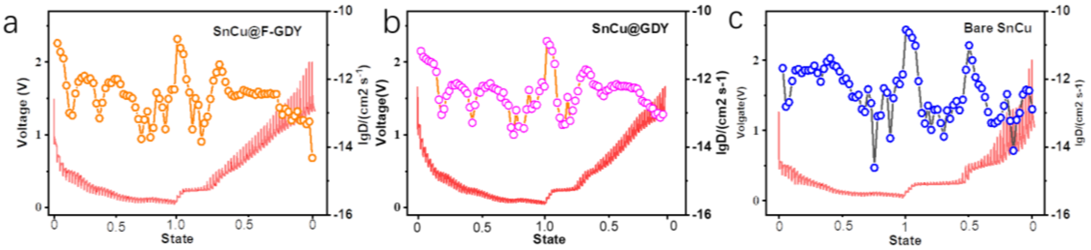

Figure S17. The GITT curves and the corresponding Na^{+} diffusion coefficient in the a) SnCu@GDY and b) bare SnCu electrode.

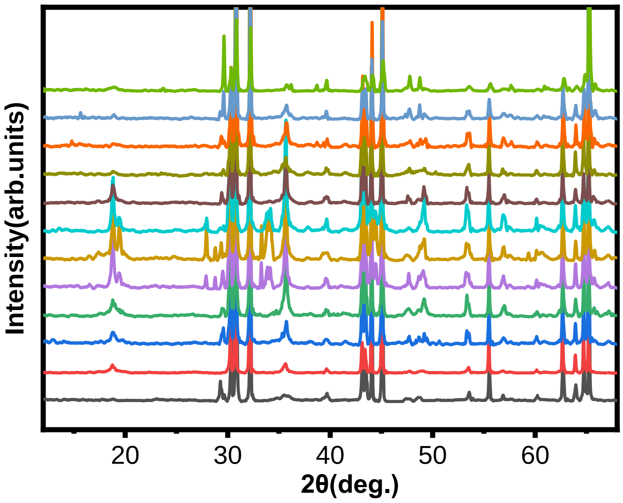

Figure S18. The ex-situ XRD of the bare SnCu in the charge and discharge process.

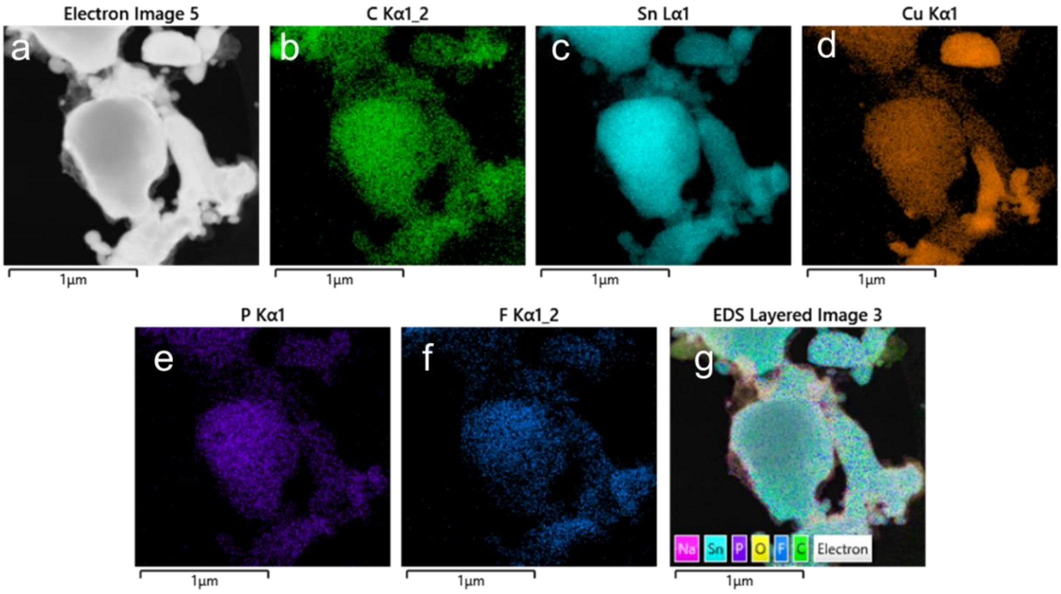

Figure S19. The elemental mapping images of the bare SnCu electrode after 50 cycles.

The images clearly show the coarsening phenomenon of the bare SnCu in the cycling. The size of the particles is increased from 100 nm to about 1 μm. The enrichment of Cu elements on some particles is clearly seen. The enrichment of Cu elements and the coarsening phenomenon demonstrate the reassembly and segregation of the electrode during cycling without the physical barrier on the surface.

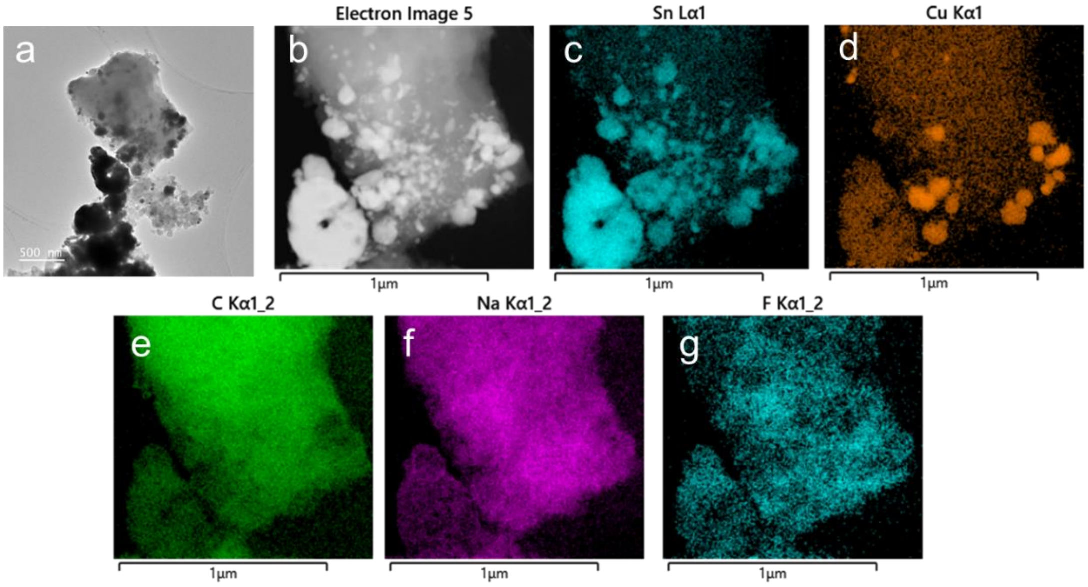

Figure S20. The elemental mapping images of the SnCu@GDY electrode after 50 cycles.

The elemental mapping of the SnCu@GDY reveals that the GDY protection can

partly restrain the pulverization and the coarsening of SnCu nanoparticles. In the GDY network, the coarsening of SnCu is efficiently stopped. However, the spheric morphology of SnCu nanoparticles is completely disappeared. Once the GDY protection layer on the SnCu is broken, the coarsening phenomenon is clearly observed. The broken of the GDY protection layer on the nanoparticle is ascribing to the extremely large volume strain of 420 % of Sn for storing the Na^+, which is far beyond the limited elongation of the GDY 7 %. Therefore, the destroyed GDY interface cannot provide consistent protection against the pulverization and the coarsening.

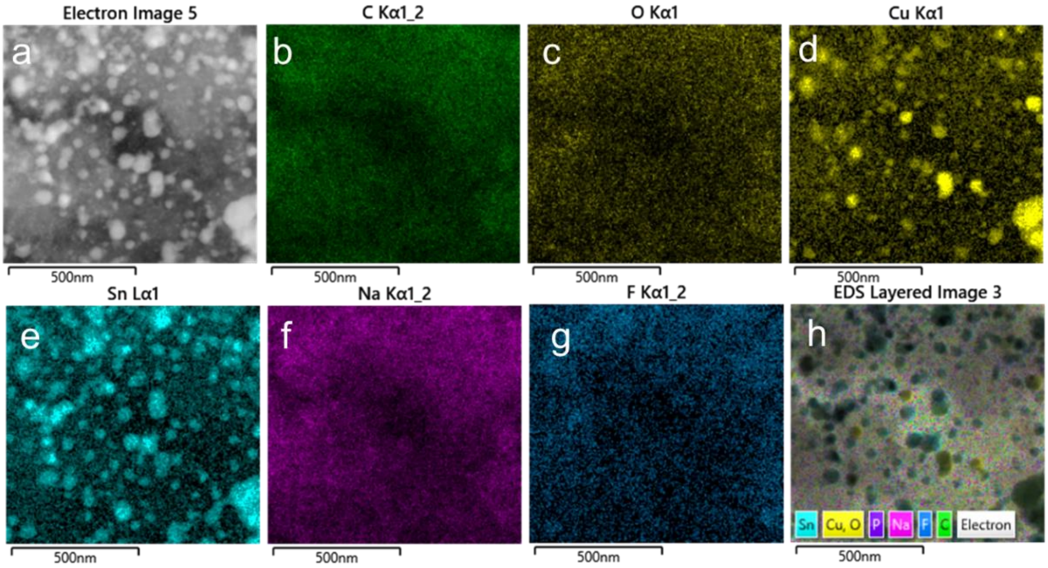

Figure S21. The elemental mapping images of SnCu@F-GDY electrode after 50 cycles.

It can be seen that SnCu nanoparticles are well dispersed in the F-GDY. The elemental mapping images clearly show the spheric morphology of the SnCu nanoparticles in the F-GDY network. Because the F-GDY can have self-adaptive character against the dynamic change of SnCu particles, the protection from F-GDY is more stable than the GDY. Thus, the pulverization and coarsening are efficiently stopped.

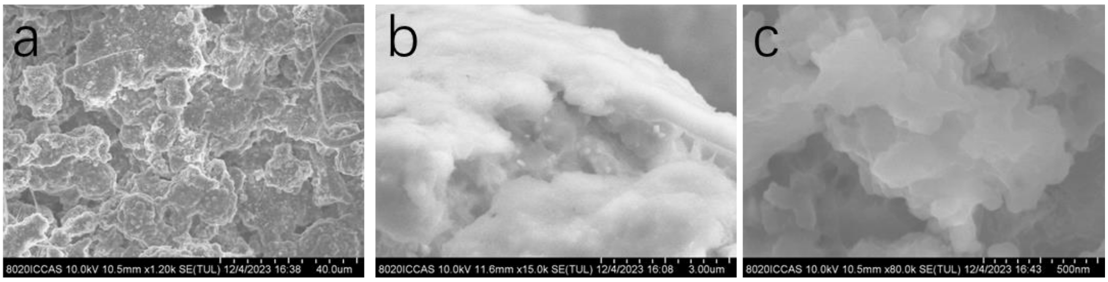

Figure S22. The SEM image of the bare SnCu after 100 cycles.

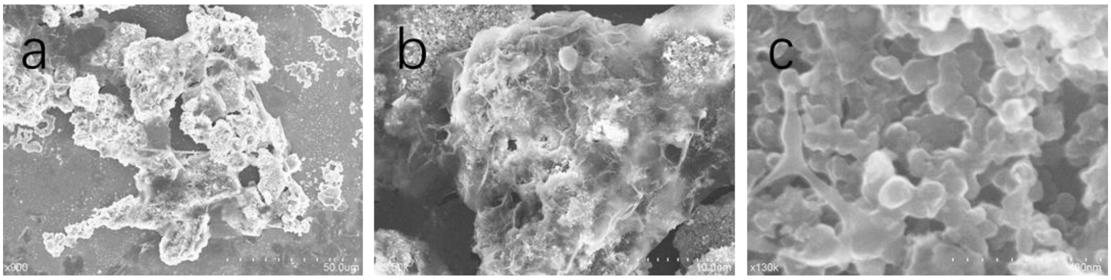

Figure S23. The SEM image of the SnCu@GDY after 100 cycles.

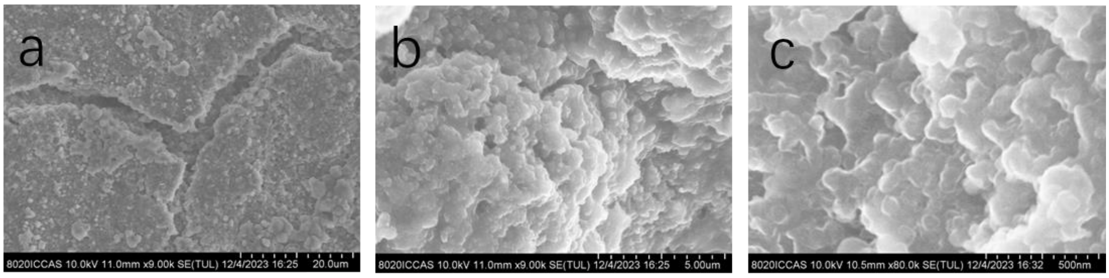

Figure S24. The SEM image of the SnCu@F-GDY after 100 cycles.

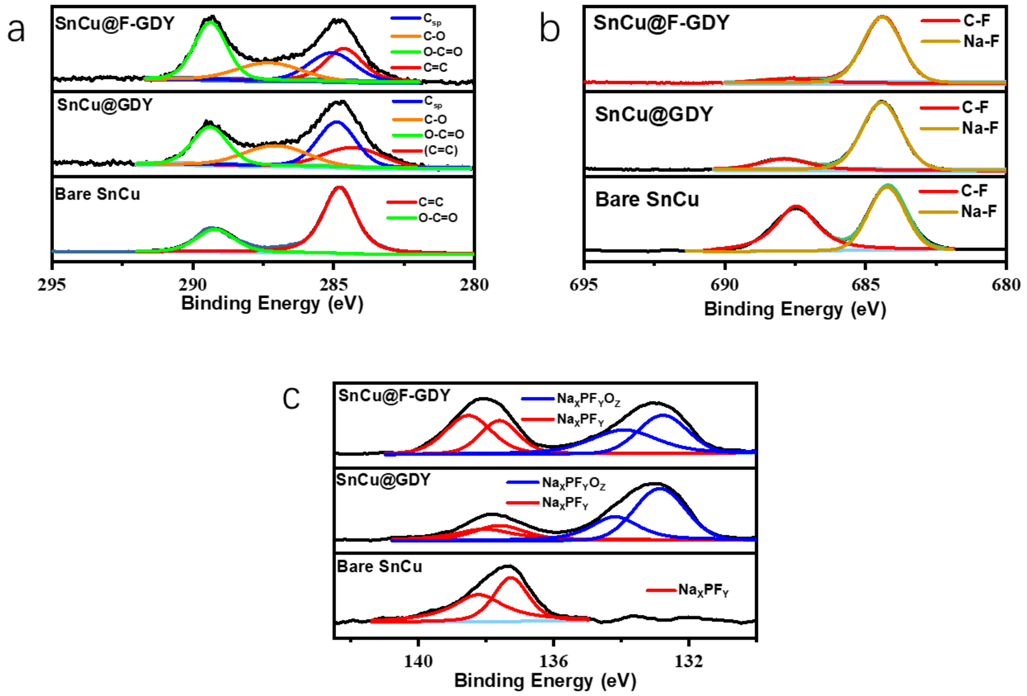

Figure S25. The high resolution XPS spectra of the electrodes after cycling.

1. Li, G.; Li, Y.; Liu, H.; Guo, Y.; Li, Y.; Zhu, D., Architecture of graphdiyne nanoscale films. *Chem. Commun.* **2010,** *46* (19), 3256-3258.
